# Cytokine-induced killer cell delivery enhances the antitumor activity of oncolytic reovirus

**DOI:** 10.1371/journal.pone.0184816

**Published:** 2017-09-18

**Authors:** Xing Zhao, Weiwei Ouyang, Cariad Chester, Shiqi Long, Nianxue Wang, Zhixu He

**Affiliations:** 1 Stem Cell and Tissue Engineering Research Center, Guizhou Medical University, Guiyang, Guizhou, China; 2 Department of Immunology, Guizhou Medical University, Guiyang, Guizhou, China; 3 Department of Thoracic Oncology, Affiliated Hospital of Guizhou Medical University, and Guizhou Cancer Hospital, Guiyang, Guizhou, China; 4 Department of Medicine, Division of Oncology, Stanford University, Stanford, California, United States of America; Swedish Neuroscience Institute, UNITED STATES

## Abstract

Oncolytic viruses (OV) have recently emerged as a promising therapeutic modality in cancer treatment. OV selectively infect and kill tumor cells, while sparing untransformed cells. The direct cytotoxic effects combined with the capacity to trigger an immune response make OV an appealing combination partner in the burgeoning field of cancer immunotherapy. One of the leading OV therapeutic candidates is the double-stranded RNA virus reovirus. In order to improve the oncolytic activity of reovirus and allow for systemic administration despite the prevalence of neutralizing antibodies, cytokine-induced killer (CIK) cells were explored as cell carriers for reovirus delivery. In this study, CIK cells were successfully loaded with reovirus *ex vivo*, and viral replication was limited in CIK cells. Confocal microscopy and flow cytometry demonstrated that CIK cells retained reovirus on the surface. Moreover, CIK cells could promote reovirus infection of tumor cells in the presence of neutralizing antibodies; meanwhile, cytotoxicity of CIK cells was increased after loading with reovirus. These findings support further investigation of reovirus and CIK combination for antitumor therapy.

## Introduction

The last decade has witnessed exciting breakthroughs in a novel treatment paradigm for cancer: oncolytic virotherapy. Oncolytic viruses (OV) are viruses that selectively grow, replicate within, and kill tumor cells while leaving untransformed cells unharmed [1]. The Respiratory Enteric Orphan virus, or reovirus, is one of the leading OV in therapeutic development (REOLYSIN®, Oncolytics Biotech Inc.) [2]. Reovirus therapy is in phase I, phase II, and phase III clinical trials in several countries, and has been assessed in combination with a variety of established treatment modalities, achieving encouraging clinical results [3–6]. However, the full efficacy of reovirus therapy may be limited by the presence of neutralizing anti-reoviral antibodies (NARA).NARA bind the virus and subsequently block viral attachment to cellular surface receptors, inhibiting viral infection and replication. NARA are found in most adults since reovirus is ubiquitous in the environment. Immunologic analysis of patients enrolled in reovirus clinical trials has consistently detected circulating NARA [4, 5, 7, 8]. This humoral immune response presents a significant barrier to intravenously administered reovirus reaching, colonizing, and eliminating disseminated tumor beds.

The advent and development of cell carriage strategies for virotherapy presents a potential solution to the circulating NARA problem. Cell carriage refers to the capacity of some OV to be “carried” by immune cells to the tumor. Reovirus was previously shown to infect and utilize peripheral blood mononuclear cells (PBMCs) to reach tumors [9]. Combination of reovirus with cytotoxic adoptive cell therapy (ACT) offers a promising anticancer therapeutic strategy. ACT is a therapeutic strategy where *in vitro*-stimulated and expanded autologous or allogeneic lymphocytes are reinfused into a patient to induce tumor regression [10, 11]. A variety of immune effector subsets have been used in ACT, including T cells transduced with chimeric antigen receptors, tumor-infiltrating T (TIL) cells, and lymphokine-activated killer (LAK) cells.

Recently, another immune-activated subset, cytokine-induced killer (CIK) cells, have shown promising antitumor activity. CIK cells are developed from PBMCs and show tumor-lytic properties *in vitro* and *in vivo* [12]. CIK cells are prepared *ex vivo* by stimulating PBMCs with a cocktail of interferon-gamma (IFN-γ), an anti-CD3 monoclonal antibody (OKT3), and interleukin-2. CIK cells are cytotoxic to a variety of tumor targets and demonstrate superior *in vivo* antitumor activity compared with LAK cells [13]. In the last decade, multiple clinical studies have established the safety and efficacy of CIK cells in a broad range of solid and hematologic malignancies [14–17]. CIK cells have previously been shown to provide cell carriage to a modified vaccinia virus in both immunodeficient and immunocompetent mouse models of ovarian cancer [18]. In this study, we tested the feasibility of using CIK cells as a protective delivery vehicle to carry oncolytic reovirus to the tumor, avoiding antibodies neutralizing.

## Material and methods

### Cell lines and virus

The murine fibroblastic cell line L929 was obtained from the American Type Culture Collection (ATCC) and cultured in Dulbecco’s modified eagle’s medium (DMEM; Gibco) supplemented with 10% fetal bovine serum (FBS; Gibco), 1% (v/v) glutamine (Gibco) and 1% (v/v) penicillin/streptomycin (Gibco). The prostate cancer cell line PC-3, colorectal carcinoma cell line DLD-1, and large cell lung carcinoma cell line NCI-H460 were obtained from China Center for Type Culture Collection (CCTCC), and cultured in RPMI-1640 at 37°C, 5% CO2 with 95% humidity.

Reovirus type 3 Dearing strain was obtained from ATCC (VR-824) and stored in -80°C until use. Reovirus was propagated in L929 cells, titrated by a standard plaque assay. For generation of UV-inactivated reovirus, reovirus in PBS were exposed to UV light (shortwave 254nm) for 30 minutes. The UV-induced loss of reoviral replicability was confirmed with L929 cell viability assay.

### Flow cytometry

To assess junctional adhesion molecule-A (JAM-A) expression, cells were stained with FITC-conjugated anti-JAM-A mAb (clone 1H2A9, Santa Cruz Biotechnology). In experiments evaluating reovirus to CIK cell attachment, reovirus treated CIK cells were incubated with anti-reovirus–σ3 primary antibody (1:100, 4F2; DSHB, University of Iowa, Department of Biology, Iowa City, IA, USA) at 4°C overnight. This was followed by incubation with FITC-goat anti-mouse IgG (1:100, Jackson ImmunoResearch, Inc.) secondary antibody for 30 min at 4°C. The cells were subsequently washed and stained with APC-conjugated mouse-anti-human CD3(SK7), PE-conjugated mouse-anti-human CD8(RPA-T8) or PE-conjugated mouse-anti-human CD56(MY31) antibodies, respectively. Appropriate FITC, PE, or APC isotype control antibodies were used as negative controls; all antibodies were obtained from BD Biosciences, and used according to the manufacturer’s instructions. Stained cells were analyzed on a FC500 flow cytometer (Beckman Coulter), with data analyzed with FlowJo software (Tree Star, Ashland, OR, USA).

### Generation of CIK cells and loading with reovirus

The study protocol was approved by the Ethics Committee of Guizhou Medical University, and all participants provided written informed consent. PBMCs were isolated from peripheral blood from healthy donors, by density gradient centrifugation with Ficoll-Hypaque (GE Healthcare Life Sciences; Milan, Italy). CIK cells were generated from PBMCs as previously described [19]. Briefly, PBMCs were cultured in GT-T551 medium (Takara Bio Inc.) containing 1000 U/ml human interferon γ (PeproTech) for 24 hours. PBMCs were then stimulated with 100 ng/ml anti-CD3 antibody (R&D) and 500 U/ml rHuIL-2 (PeproTech). Fresh medium containing 500 U/ml rHuIL-2 was added every 3 days. To assess CIK cell quality, aliquots of cells were harvested after 14–16 days of incubation, and characterized by phenotypic analysis and cytotoxicity assay. The major effector cells were NKT (CD3^+^CD56^+^NKT ≥ 20%) and CTL cells (CD3^+^CD8^+^CTL ≥ 60%).

To establish the optimal reovirus loading condition, CIK cells were infected with reovirus of 1 plaque forming units (pfu)/cell at both 4°C or 37°C for 2 or 4 hours, respectively. CIK cells were then washed with PBS to remove unbound viruses, and reovirus binding on CIK cells was assessed by flow cytometry. Optimal reovirus loading occurred at an MOI of 1 at 4°C for 2 hours, and this condition was used in subsequent experiments.

### Cell viability assay

To determine reovirus-mediated cell lysis, tumor cell lines (DLD-1, PC-3, and NCI-H460) and CIK cells were seeded in 96-well plates at a density of 5×10^3^ cells per well in 100μl medium and incubated overnight. After 12 hours, cells were treated with reovirus in the amounts of 0, 0.001, 0.01, 0.1 and 1 pfu/cell. After 2 hours of incubation, cells were washed with PBS to remove unbound viruses, and fresh growth medium was added. Cell viability was measured with Cell Counting Kit-8 (CCK-8, Dojindo Lab, Tokyo, Japan) following the manufacturer's instructions, and calculated as percentage of viable cells in the reovirus-treated group versus untreated controls.

### Quantitative real-time PCR

Total RNA was extracted from DLD-1, PC-3, NCI-H460, and CIK cells at 12, 24, 48, and 72 hours after infection with 1pfu/cell reovirus, respectively, with TRIZOL reagent (Invitrogen). First strand cDNA synthesis was performed using iScript cDNA Synthesis Kit (Bio-Rad Laboratories, Hercules, CA) with 0.5μg total RNA per 10μl reaction. Gene expression was quantified by real-time quantitative RT-PCR with iQSYBR Green supermix (Bio-Rad Laboratories, Hercules, CA); detection was performed by measuring the binding of the fluorescence dye SYBR Green I to double-stranded DNA. The primer sets were provided by Generay Biotechnology (see Table 1). The comparative Ct method was used for data analysis. Relative quantity was defined as 2^−ΔΔCt^, with β-actin used as the reference gene.

**Table 1:** Primers used in qRT-PCR. Gene short names, Genbank accession numbers, PCR primer sequences and amplicon length are shown.

Gene	Accession No.	Forward primer (5^’_^3^’^)	Reverse primer (5^’ _^ 3^’^)	Amplicon length(bp)
T3D	M27262	TGATTTCCATTACTTCTGCTGCTT	TCCTGTTCACGATTCCATCAGAT	72
β-Actin	NM-001101	GCCGGGACCTGACTGACTAC	TTCTCCTTAATGTCACGCACGAT	100

### Confocal microscopy

Tumor or CIK cells were seeded at a density of 2×10^4^ cells/well into glass bottom dishes, and incubated overnight at 37°C. Then, the medium was removed, and 1 pfu/cell of reovirus was incubated with tumor or CIK cells at 4°C for 2 hours. Virus inocula were then aspirated, and cells were cultured in fresh media for 12 hours. To prepare the cells for microscopy, reovirus-infected cells were washed twice with PBS, and fixed with 4% paraformaldehyde. Then, mouse anti-reovirus–σ3 primary antibody (4F2, 1:100) was applied at 4°C and incubated overnight. Afterwards, the cells were washed three times with PBS, and FITC-conjugated goat anti-mouse IgG secondary antibodies (1:50, Jackson ImmunoResearch, Inc.) were applied in 3% BSA for 30 min at 4°C. Counterstaining was performed with 4′, 6-diamidino-2-phenylindole (DAPI). Confocal images were acquired on a confocal microscope (Olympus, FV1000, Olympus Optical, Japan) in sequential scanning mode.

### Transmission electron microscopy

DLD-1, PC-3, NCI-H460, and CIK cells were incubated with 1 pfu/cell of reovirus at 4°C for 2 hours. Then, the cells were washed with PBS twice and incubated at 37°C for 12 hours. Following incubation, the cells were harvested and fixed in 2.5% glutaraldehyde in 0.1M sodium phosphate buffer (pH 7.4) at room temperature for 2 hours. The cells were then washed once in sodium phosphate buffer and dehydrated using a graded series of ethanol. After dehydration, the samples were subjected to two changes of propylene oxide and embedded in epoxy resin. After ultra-microtome sectioning, sections were analyzed on a FEI Tecnai G2 20 TWIN electron microscope operating at 200 kV.

### Reovirus hand-off in the presence of human AB serum

Human AB serum was collected from 4 healthy donors. The neutralizing effect of human AB serum was confirmed with the L929 cell line.

DLD-1, PC-3, and NCI-H460 tumor cells were used as target cells; they were kept in the log phase and passaged the day before the cytotoxicity assay. For the CIK cell transfer reovirus-mediated cytotoxicity assay, cell viability was assessed by CCK-8 assay. Briefly, DLD-1, PC-3 and NCI-H460 cells were seeded into 96-well plates at 8 ×10^5^/ml in 100μl per well for 12 h. Then, CIK cells were re-suspended at 2×10^6^ cells/ml and incubated with reovirus or UV-inactivated reovirus at 1 pfu per cell at 4°C for 2 hours and washed 3 times with PBS to remove unbound viruses. CIK cells loaded with reovirus were co-cultured with tumor cells at an E/T ratio of 10:1 in the presence of 7.5% human AB serum for 2 hours. Afterwards, CIK cells were washed, and adherent tumor cells were cultured for an additional 24, 48, and 72 hours, respectively. Cell viability was measured by CCK-8 assay according to manufacturer’s instructions. Calculate the percentage of cell death as follows: (1-A_test_ /A_control_) ×100, where A_tes_t is the absorbance of experimental wells and A_control_ is the absorbance of control wells. All experiments were performed in biological triplicate.

### Cytotoxicity assay

For the reovirus-loaded CIK cell mediated cytotoxicity assay, the CytoTox 96® Non-Radioactive Cytotoxicity Assay kit (Promega; Madison, WI) was used to quantify LDH release following the manufacturer’s protocol. Briefly, CIK cells were resuspended at 2 × 10^6^ cells/ml, and incubated with reovirus at 1 pfu per cell at 4°C for 2 hours. Then, the cells were washed twice to remove the unbound reovirus. Using an E/T ratio of 20:1, reovirus-loaded CIK cells (1.6 ×10^6^) were plated into 96-well plates, and mixed with tumor cells (8 ×10^4^) in the presence of 7.5% human AB serum (v/v). The experiment was performed in triplicate. After incubation at 37°C in 5% CO_2_ for 6 hours, culture plates were centrifuged at 250×g for 5 minutes, and 10μl of supernatant from each well was collected and analyzed by measuring the optical density (OD) value to estimate cell death. OD values were converted into percent specific cytotoxicity (%) as: (OD_Experimental_−ODEffector spontaneous−OD_Target spontaneous_)/(OD_Target maximum_–OD_Target spontaneous_) ×100.All assays were performed in triplicate, with CIK cells isolated from 3 unique donors.

### Statistical analysis

GraphPad Prism version 6 (GraphPad, San Diego, CA) was used for statistical analysis. For normally distributed variables, comparisons between two given groups were performed by the parametric Student's *t* test; multiple group comparisons were carried out by one-way or two-way analysis of variance (ANOVA).

## Results

### Reovirus exerts an oncolytic effect on tumor cells but not on CIK cells

**Fig 1:**
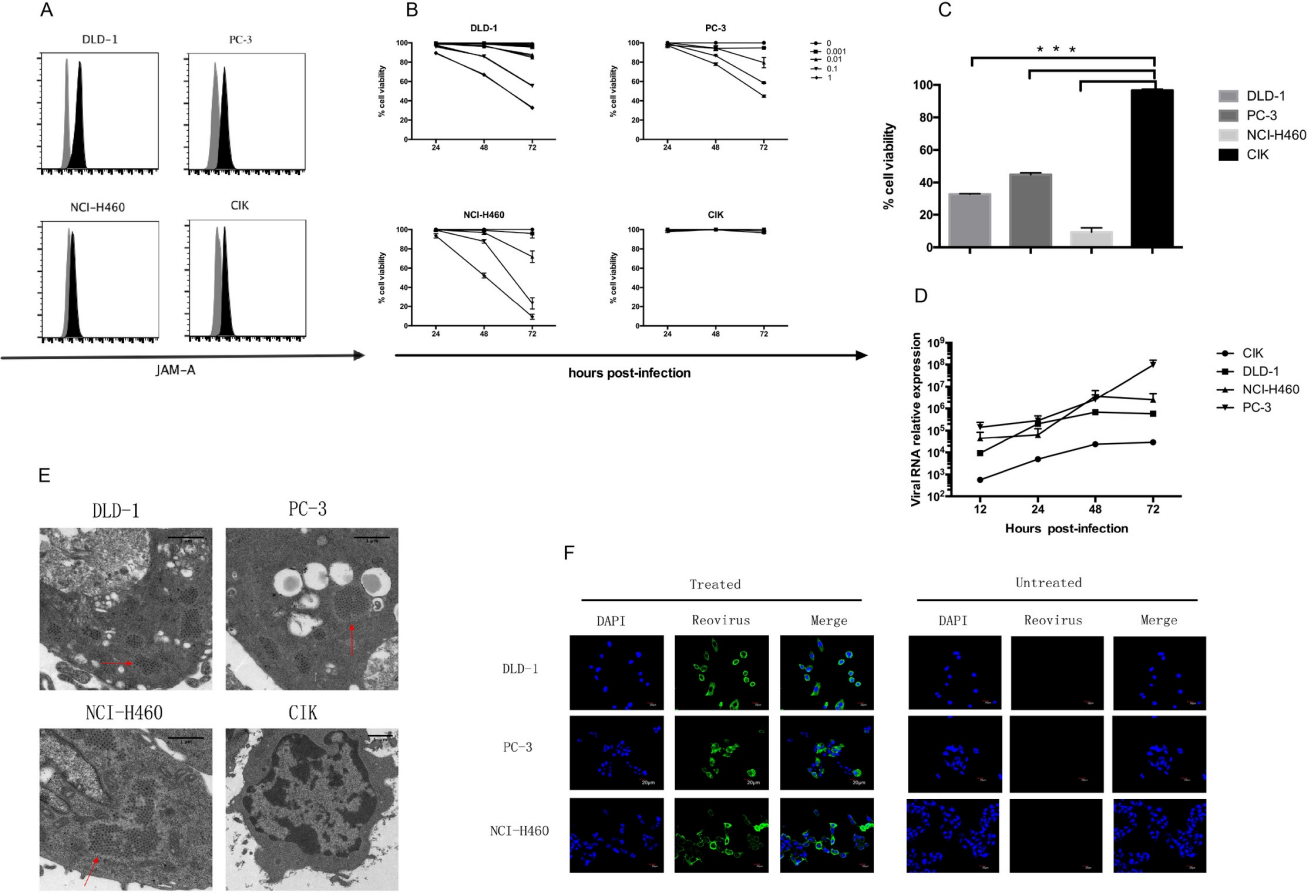
Reovirus exerts oncolytic effects on tumor cells but not on CIK cells. (A) JAM-A surface expression on tumor (DLD-1, PC-3, and NCI-H460) and CIK cells. Flow cytometry was performed with FITC-conjugated mAb targeting JAM-A. Grey and black histograms represent cells treated with FITC-conjugated isotype-matched- and specific FITC-conjugated mAbs, respectively. (B) Oncolytic effect of reovirus on tumor cell lines and CIK cells. DLD-1, PC-3, NCI-H460, and CIK cells were seeded in 96-well plates and infected with reovirus at MOIs ranging from 0.001 to 1 pfu/cell for 24 h, 48 h and 72 h, respectively. Cell viability was assessed by CCK-8 assay. Data are expressed as percentages of cell viability, and represent the means (with SD) of triplicate values. (C) Cell viability of DLD-1,PC-3, NCI-H460, and CIK cells after infection with reovirus at a MOI of 1 for 72 hours. All values reported as means (with SD) of three independent experiments. ***P<0.01. (D) Real-time PCR analysis of reovirus gene expression in tumor and CIK cells at 12, 24, 48 and 72 hours after reovirus infection. Tumor cells and CIK cells were incubated with 1pfu/cell reovirus for 2 h. Fresh medium was added after two washing, and cells were incubated at 37°C for the indicated times. Data are mean values of three samples expressed as fold increase compared to non-infected controls, using the 2^-ΔΔCt^ method. (E) Detection of viral particles in reovirus infected cells by transmission electron microscopy. DLD-1, PC-3, NCI-H460, and CIK cells were infected with reovirus at a MOI of 1 for 12 hours. The uncoated viruses were washed off, and the samples were fixed for assessment by thin-section electron microscopy. Arrows indicate large virion inclusions. Scale bar, 1μm. (F) Internalization of reovirus by DLD-1, PC-3 and NCI-H460 cells. Tumor cells were absorbed with 1 pfu/cell reovirus at 4°C for 2 hours. The inoculum was removed, and cells were incubated in fresh medium for another 12 hours. The virus was washed off, following by DAPI (blue) and reovirus (green) staining; samples were imaged by confocal microscopy. Scale bar, 20μm.

For a successful cell carriage strategy, OV must infect both the carrier and tumor cells, while only damaging the tumor cells. To measure the susceptibility of CIK and tumor cells to reovirus-induced oncolysis, we assessed the expression of JAM-A, the main cellular receptor for reovirus. JAM-A was consistently expressed on CIK cells and human tumor cell lines, including DLD-1, PC-3 and NCI-H460 (Fig 1A). To determine if reovirus infection was deleterious to CIK cells and/or tumor cells, we assessed cell viability of CIK and tumor cells after incubation with reovirus (Fig 1B). Cells were infected with reovirus at multiplicity of infection (MOI) values ranging from 0.001 to 1 pfu per cell, and percentages of cell viability were determined at 24, 48, and 72 hours post infection, respectively. Three tumor cell lines were highly sensitive to virus-mediated lysis, while reovirus showed no significant lytic effects on CIK cells within 72 hours (Fig 1C).

To confirm that increased cell lysis was due to viral replication, we then infected cells with reovirus at 1 pfu/cell and measured virus gene expression (Fig 1D). Reovirus replication was observed in all tested tumor cell lines, and corresponded to the increase of tumor cell lysis. In contrast, viral replication was limited in CIK cells, and cell viability data suggested only a minor lytic effect of reovirus on CIK cells. To confirm that reovirus selectively targets and damages tumor cells, we analyzed the cytoplasmic accumulation of viral particles in tumor cells by electron microscopy and confocal microscopy. There were no viral particles in the cytoplasm of reovirus-infected CIK cells, while numerous virion clusters were observed in the cytoplasm of tumor cells, which also exhibited large amounts of vacuoles in the cytoplasm (Fig 1E). Green fluorescence accumulation in the cytoplasm of tumor cells was observed by confocal microscopy (Fig 1F).

### CIK cells provide cell carriage to reovirus

**Fig 2:**
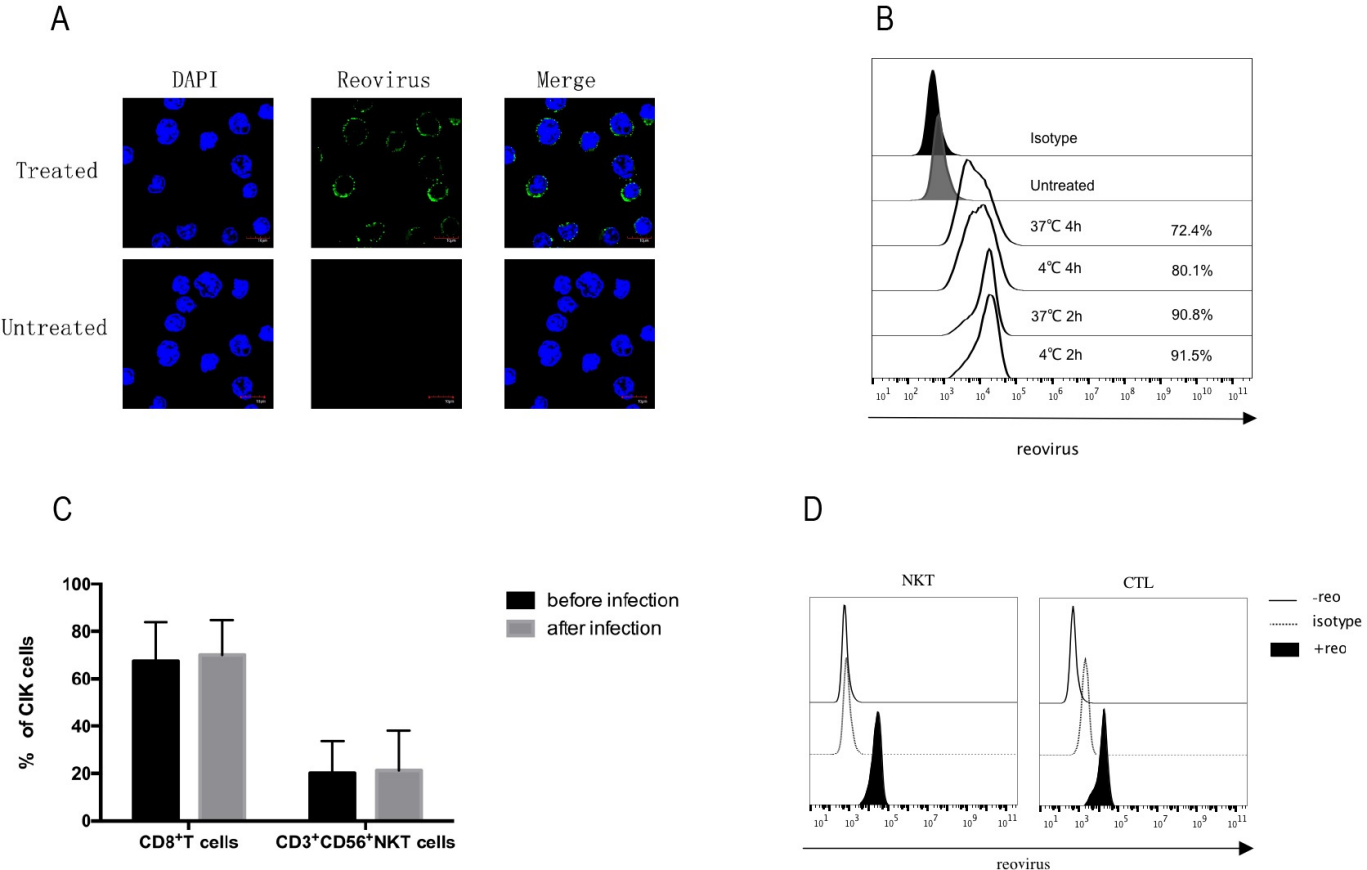
Reovirus loading onto CIK cells. (A) Confocal microscopy detected reovirus loading on CIK cell surface. CIK cells were infected with 1 pfu/cell reovirus at 4°C for 2 hours. The inoculum was removed, and CIK cells were incubated in complete growth medium for another 12 hours. The uncoated viruses were washed off; after DAPI (blue) and reovirus (green) staining, imaging was performed by confocal microscopy. Scale bars,10μm. (B) Optimizing the conditions for reovirus loading onto CIK cells. CIK cells were infected with 1 pfu/cell reovirus at 4°C or 37°C for 2 hours or 4 hours, respectively. The unbound viruses were washed off, followed by staining with anti-reovirus–σ3 primary antibody and FITC-goat anti-mouse IgG secondary antibody; reovirus binding on CIK cells was assessed by flow cytometry. Black filled and grey filled histograms represent isotype and untreated control staining, respectively, whereas open-black line histograms represent cells stained with specific FITC-conjugated mAb. (C) Flow cytometry analysis of CIK cell phenotypes before and after reovirus infection. CIK cells were infected with or without 1 pfu/cell reovirus at 4°C for 2 hours, and stained with anti-CD3-APC, anti-CD8-PE or anti-CD56-PE for flow cytometry. (D) Assessment of reovirus binding to CD8^+^CTL and CD3^+^CD56^+^NKT cells. CIK cells were infected with 1pfu/cell reovirus at 4°C for 2 hours, washed twice, and stained with anti-reovirus–σ3 primary antibody at 4°C overnight, cells were than washed and stained with FITC-goat anti-mouse IgG secondary antibody, anti-CD3-APC, anti-CD8-PE or anti-CD56-PE antibodies, before acquisition using flow cytometry.

Having demonstrated that CIK cells avoid the deleterious consequences of viral infection, we next confirmed reovirus binding onto CIK cells by confocal microscopy, which indicated the presence of reovirus on the surface of CIK cells (Fig 2A). Viral particles remained on CIK cell surface and did not enter the cytoplasm, even after a 12-hour incubation with 1 pfu/cell of reovirus. The optimum reovirus loading conditions should have maximal virus binding and minimal CIK cell death. We then identified the optimal conditions for loading CIK cells with reovirus particles by incubating CIK cells with 1 pfu/cell reovirus at 4°C or 37°C for 2 hours or 4 hours, respectively. Maximum reovirus binding to CIK cells was achieved after incubation at 4°C for 2 hours (Fig 2B). Interestingly, we found that prolonged incubation durations and increased temperatures both decreased reovirus binding. Taken together, these data confirmed the optimal conditions for *ex vivo* loading to be incubation of reovirus with CIK cells at an MOI of 1 at 4°C for 2 hours. The phenotypic changes of CIK cells during reovirus infection were also characterized to ensure reovirus-infection did not bias CIK cells towards a single phenotype. There were no differences in the percentages of CD8^+^ T cells and CD3^+^CD56^+^ NKT cells in CIK cells before and after reovirus exposure (Fig 2C). We then explored which subpopulations of CIK cells were responsible for reovirus binding. Both CTL cells and NKT cells bound and retained reovirus on the surface (Fig 2D).

### CIK cells efficiently transport reovirus to the tumor cells in the presence of human AB serum

**Fig 3:**
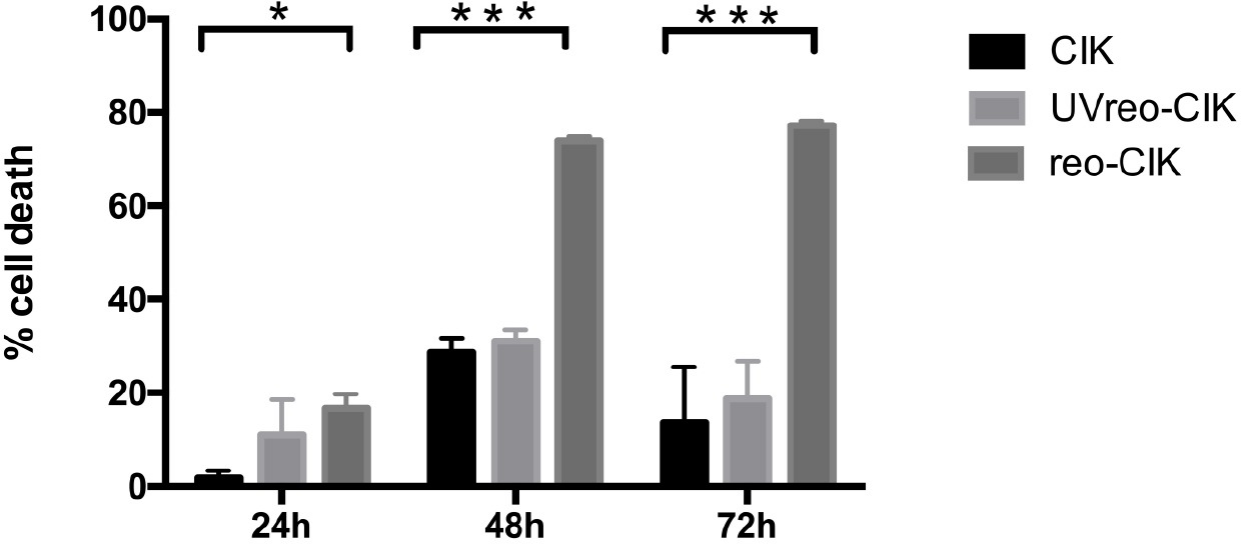
CIK cells deliver reovirus to tumor cells even in the presence of human AB serum. CIK cells were pre-incubated with 1 pfu/cell live or UV-inactivated reovirus at 4°C for 2 hours, washed twice, and added to PC-3 cells (8×10^4^) at 10:1 ratio in the presence of 7.5% human AB serum. After 2 hours of incubation, CIK cells were washed off with fresh medium, and adherent tumor cells were incubated for additional 24-, 48- and 72-hours, respectively. Cell viability was assessed by the CCK-8 assay at the indicated time points. Representative experiments from three replicates are shown. Error bars represent standard deviation. (* *P*<0.05, ***P<0.01)

Next, the ability of CIK cells to deliver reovirus to tumor cells in the presence of preexisting neutralizing antibodies was investigated. Human AB serum can neutralize reovirus and eliminate reovirus-mediated oncolysis, the effect of reovirus-induced L929 cells lysis was effectively abolished by 7.5% human AB serum (S1 Fig), we therefore used 7.5% human AB serum in the subsequent blocking experiments. CIK cells were infected with live or UV-inactivated reovirus at 1 pfu/cell for 2 hours, and added to plated tumor cells. To exclude the possibility of CIK cell induced cytotoxicity, CIK cells were removed after 2-hours of incubation. Adherent tumor cells were cultured for an additional 24, 48 and 72 hours, respectively, and tested for cell viability. At the 24-, 48- and 72-hour time points, the tumor cells exposed to reovirus-loaded CIK cells showed significantly increased cell death compared with those exposed to CIK cells alone and UV-inactivated reovirus-loaded CIK cells (Fig 3). This finding suggested that reovirus had been transferred from CIK cells to tumor cells during the initial 2 hours of incubation and still retained its oncolytic capacity after CIK cell carriage to the tumor, even in the presence of human AB serum. Importantly, the oncolytic effect was reovirus replication dependent, because cytotoxicity of UV-inactivated reovirus pre-treated CIK cells was equivalent to that of CIK cells alone.

### Reovirus infection enhances CIK cell cytotoxicity

**Fig 4:**
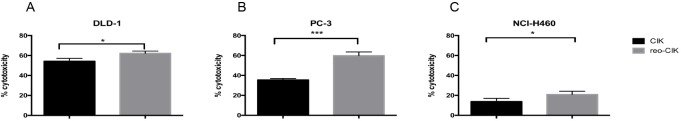
Cytotoxicity of reovirus-loaded CIK cells toward tumor cell lines. CIK cells were loaded with 1 pfu/cell reovirus at 4°C for 2 h. DLD-1, PC-3 and NCI-H460 tumor cells were used as target cells. Cytotoxic activity was measured at 6 h by LDH release assay performed on target cells at an effector-to-target ratio of 20:1. The killing assay was performed with CIK cells only (filled black) and reovirus-loaded CIK cells(filled grey) with 7.5% human AB serum. Data are expressed as mean percentage (and SD) of 3 independent experiments. *P<0.05, ***P<0.01.

We then set to measure the cytotoxicity of reovirus pre-treated CIK cells co-cultured with tumor cells in the presence of 7.5% AB serum. Interestingly, reovirus pre-treated CIK cells could enhance cytotoxicity even in the presence of neutralizing antibodies. Tumor cell lines were placed in contact with reovirus pre-treated CIK cells at 20:1 E/T ratios, and target cell killing was measured after 6 hours by the LDH release assay. As shown in Fig 4A–4C, CIK cells efficiently killed DLD-1, PC-3 and NCI-H460 target cell lines with 54.3±2.8%,35.1±1.5% and 13.8±3.3% cytotoxicity at 20:1 E/T ratio, respectively. Cytotoxicity of CIK cells was further increased after pre-treatment with reovirus, with 62.3±2.2%,59.8±3.7% and 20.8±3.3% cytotoxicity observed in target cell lines at the same E/T ratio, respectively.

## Discussion

Oncolytic virotherapy is a promising treatment strategy and combination partner for anticancer therapy. Talimogene laherparovec (T-Vec), a modified herpes simplex virus, garnered the first FDA approval for an oncolytic virus in 2015; following its success, over two dozen OVs have entered clinical development. However, T-Vec and many other OVs are limited to intratumoral administration, greatly reducing their therapeutic applicability. In this study, we demonstrated a novel strategy for overcoming the limitations of intravenous administration and establish the therapeutic synergy between CIK cells and reovirus. Cell carriage provides a compelling mechanism for systemic administration of reovirus and is more desirable than intratumoral delivery because it offers the potential to target metastatic disease and addresses disease burden in multiple, distinct tumor beds. Herein, we showed that CIK cell carriage strategy overcomes the limitations of neutralizing antibodies and may provide a superior approach for oncolytic virus-based antitumor therapy.

These findings demonstrated that reovirus and CIK cells are both potent antitumor agents, and superior as a combination strategy. Reovirus efficiently infected and replicated in tumor cells, causing tumor cell lysis. The unique susceptibility of cancer cells to reovirus infection is a result of defective immune response and aberrant cellular signaling that occur during tumorigenesis. With reovirus, tumor infective selectivity is thought to result from oncogenic Ras signaling and compromised antiviral defenses [20]. Ras activation enhances viral uncoating and disassembly, increases the generation of viral progeny with enhanced infectivity, and accelerates the release of progeny through enhanced apoptosis [21]. While Ras-mutant targeting by reovirus is well established, the effects of reovirus infection on CIK cells were previously unknown. As shown above, reovirus infection did not induce cytolytic effects on CIK cells, and most of reoviral particles bound to the surface of CIK cells without being endocytosed. Reovirus might be differently localized on carrier cells; CIK cells carried reovirus on the surface while DCs protect reovirus by internalization prior to delivery to tumor cells. The combination of minor lytic effects of reovirus on CIK cells, low intracellular viral replication, and strong extracellular binding indicates that CIK cells are potent candidate cell carriers for reovirus. Furthermore, survival of CIK cells remained over 95% even 3 days after infection with reovirus. The sustained viability of CIK cells is important when considering CIK cell utility in a cell carriage strategy. Prolonged CIK cell survival after reovirus infection is essential to allow trafficking and accumulation of CIK cells in the tumor beds.

Reovirus were delivered by CIK cells, intrinsically with tumor chemotaxis and killing capability, which may be a solution to the problems encountered in current clinical trials. We next explored whether CIK cells could mask reovirus particles from neutralizing antibodies and transport the virus to tumor cells. As expected, reovirus still retained oncolytic effects after transport by CIK cells even in the presence of neutralizing antibodies. The transport of reovirus from CIK cells to tumor cells further supports the cell carriage potential of CIK cells. We hypothesized that after reovirus arriving at the tumor site, reoviral infection can induce tumor lysis, provoking an antitumor immune response; the tumor cells infected by reovirus can secrete RANTES, IL-8,MIP-1α, and MIP-1 β, creating a proinflammatory milieu that elicits a chemotactic response [22]. Reovirus infection induces homing of NK cells, cytotoxic CTL and DCs to the tumor microenvironment, and increases tumor antigen expression and presentation by DCs and NK cell activation [23].

In addition to the synergy provided by CIK cell carriage, reovirus infection increased CIK cell cytotoxicity towards the tumor cells. This effect may be due to the capacity of reovirus to activate immune cells. We and others have demonstrated that reovirus treatment induces increased interferon-induced gene expression and upregulated activation markers on NK cells, and significantly enhances anti-tumor activity [24–26]. Mechanistically, we hypothesized that enhancement of NK cell cytotoxicity induced by reovirus is dependent on increased perforin and FasL, whereas others have proposed this immune activation appears to result from pathogen associated molecular pattern (PAMP) recognition. Reoviral double-stranded RNA can induce IFN-I responses via the cytoplasmic RNA sensor and PAMP receptor retinoic acid-inducible gene 1 (RIG-I). Triggering an IFN-I response after RIG-I ligation can increase the cytotoxicity of NKT cells and CTLs, the main constituent immune populations of expanded CIK cells.

In conclusion, according to the current work, CIK cells provide a compelling cell carrier for reovirus, and this combination therapy should be pursued in further preclinical testing.

## Supporting information

S1 Fig The effect of reovirus-induced L929 cells lysis in the presence of human AB serum. (TIF)

S1 Table Oncolytic effect of reovirus on tumor cell lines and CIK cells. (XLSX)

S2 Table Cell viability of DLD-1,PC-3,NCI-H460 and CIK cells after infection with reovirus at a MOI of 1 for 72 hours. (XLSX)

S3 Table Real-time PCR analysis of reovirus gene expression in tumor and CIK cells at 12,24,48 and 72 hours after reovirus infection. (XLSX)

S4 Table Flow cytometry analysis of CIK cell phenotypes before and after reovirus infection. (XLSX)

S5 Table CIK cells deliver reovirus to tumor cells even in the presence of human AB serum. (XLSX)

S6 Table Cytotoxicity of reovirus-loaded CIK cells toward tumor cell lines. (XLSX)
