# Correction: Cytokine-induced killer cell delivery enhances the antitumor activity of oncolytic reovirus

**DOI:** 10.1371/journal.pone.0192090

**Published:** 2018-01-25

**Authors:** Xing Zhao, Weiwei Ouyang, Cariad Chester, Shiqi Long, Nianxue Wang, Zhixu He

The following information is missing from the Funding section: This study was supported by the Joint Social Development Foundation from Bureau of Science and Technology of Guizhou Province and Guizhou Medical University No.[2011]011, http://kjt.gzst.gov.cn/.
