# Expression of Concern: Cytokine-induced killer cell delivery enhances the antitumor activity of oncolytic reovirus

**DOI:** 10.1371/journal.pone.0299280

**Published:** 2024-02-15

**Authors:** The PLOS ONE Editors

Following the publication of this article [1, 2], concerns were raised that the following panels in Figure 1A appear to partially overlap, despite being used to present different experimental conditions:

○ Treated DAPI and Untreated DAPI;
○ Treated Merge and Untreated Merge.

In addition, during editorial assessment of the Supporting Information files, it was noted that the files provided with the article present mean results only, and the individual-level data underlying these results have not been provided.

The authors did not respond to editorial communications regarding these concerns. In the absence of original image data, the image concerns listed above cannot be resolved. Furthermore, in the absence of the individual-level data underlying the quantitative results, this study does not meet the requirements of the PLOS Data Availability policy in place at the time of the article’s publication.

The *PLOS ONE* Editors issue this Expression of Concern to notify readers of the unresolved image and data availability concerns.
