# Supplementary figures and images for: Cytokine-induced killer cell delivery enhances the antitumor activity of oncolytic reovirus
Source: PLoS One. 2017 Sep 18;12(9):e0184816. doi: 10.1371/journal.pone.0184816 (PMC5602626; doi:10.1371/journal.pone.0184816)

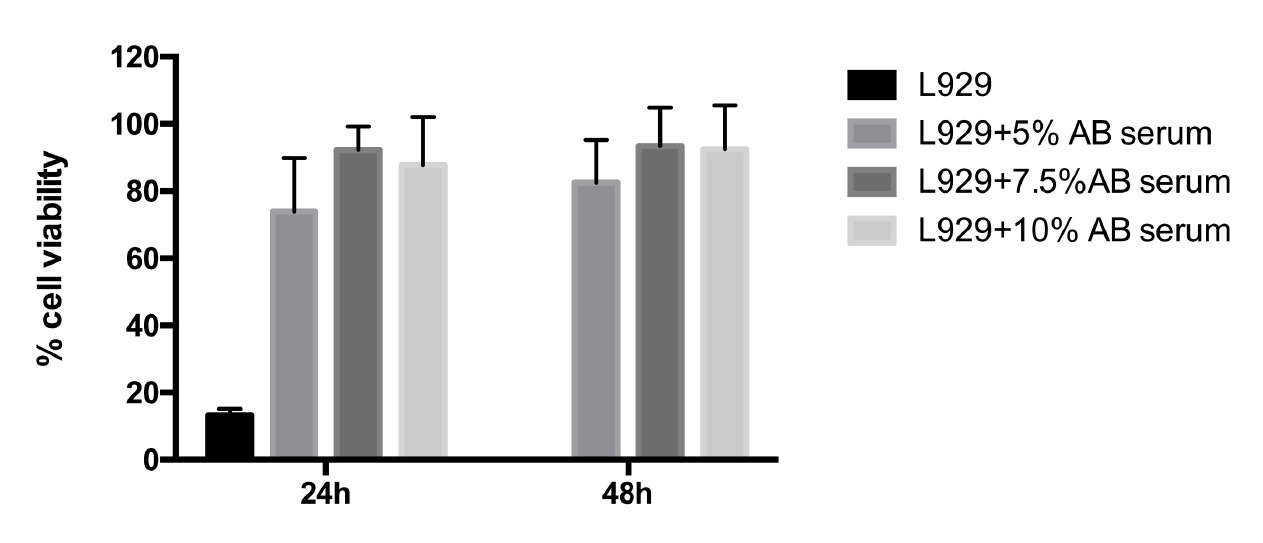

Supplement: S1 Fig — (TIF) [file pone.0184816.s001.tif]
